# Insights into the *Gryllus bimaculatus* Immune-Related Transcriptomic Profiling to Combat Naturally Invading Pathogens

**DOI:** 10.3390/jof6040232

**Published:** 2020-10-18

**Authors:** Abid Hussain, Muhammad Waqar Ali, Ahmed Mohammed AlJabr, Saad Naser AL-Kahtani

**Affiliations:** 1 Laboratory of Bio-Control and Molecular Biology, Department of Arid Land Agriculture, College of Agricultural and Food Sciences, King Faisal University, Hofuf 31982, Saudi Arabia; abhussain@kfu.edu.sa (A.H.); salkahtani@kfu.edu.sa (S.N.A.-K.); 2 Institute of Research and Consultancy, King Faisal University, Hofuf 31982, Saudi Arabia; 3 Ministry of Environment, Water and Agriculture, Riyadh 11442, Saudi Arabia; 4 Institute of Fruit and Tea, Hubei Academy of Agricultural Sciences, Wuhan 430064, China; waqar3811@gmail.com

**Keywords:** antimicrobial peptides, genomics, host defense, immunity, next generation sequencing, transcriptome, two-spotted field crickets

## Abstract

Natural pathogen pressure is an important factor that shapes the host immune defense mechanism. The current study primarily aimed to explore the molecular basis of the natural immune defense mechanism of a sporadic pest, *Gryllus bimaculatus*, during swarming by constructing cDNA libraries of the female mid-gut, male mid-gut, testes, and ovaries. The Illumina HiSeq platform generated an average of 7.9 G, 11.77 G, 10.07 G, and 10.07 G bases of outputs from the male mid-gut, female mid-gut, testes, and ovaries and libraries, respectively. The transcriptome of two-spotted field crickets was assembled into 233,172 UniGenes, which yielded approximately 163.58 million reads. On the other hand, there were 43,055 genes in common that were shared among all the biological samples. Gene Ontology analysis successfully annotated 492 immune-related genes, which comprised mainly Pattern Recognition Receptors (62 genes), Signal modulators (57 genes), Signal transduction (214 genes), Effectors (36 genes), and another immune-related 123 genes. In summary, the identified wide range of immune-related genes from *G. bimaculatus* indicates the existence of a sophisticated and specialized broad spectrum immune mechanism against invading pathogens, which provides, for the first time, insights into the molecular mechanism of disease resistance among two-spotted field crickets.

## 1. Introduction

The two-spotted field cricket, *Gryllus bimaculatus* (Orthoptera, Gryllidae, Gryllinae), is becoming a popular model insect in order to explore behavioral adaptations [1,2], evolutionary biology [3], and physiological [4] and developmental mechanisms [5,6]. The acceptance of two-spotted field cricket for research has mainly occurred because of its widespread abundance in different geographical regions, especially in Asia, Africa, and Europe, in addition to its ease of rearing under laboratory conditions. Furthermore, two-spotted field crickets are currently praised for their possible contribution to global food security by providing an alternate source of protein due to their intrinsic ability to efficiently utilize water and feed compared to traditional livestock [7]. In this regard, a recent study has successfully grown two-spotted field crickets on the by-products of the food industry in order to achieve the targets for the circular economy [7].

The *G. bimaculatus* is categorized as a sporadic pest due to its occasional outbreak under special circumstances at a specific time of the year. In the Kingdom of Saudi Arabia, the outbreak of two-spotted field crickets (black crickets) made its way to such western regions of the country as Makkah and Madinah, where it invaded human dwellings, including places of worship, in which chemical control approaches are not feasible. The residents of the regions were perplexed during their swarming in the last quarter of 2018 till the first quarter of 2019. The latest surveys (unpublished) revealed changing climatic conditions and vegetation covers providing an enormous breeding opportunity for their reproductive success to build sporadic outbreaks of two-spotted field crickets, especially in the Kingdom of Saudi Arabia.

The management of such sporadic outbreaks has become very challenging in urban areas, as synthetic chemical pesticides cannot be applied due to potential threats to public health. Therefore, the use of effective biocontrol agents, which can quickly overcome the host immune defense mechanism, are gaining special attention [8,9]. In the meantime, the host has evolved a highly specialized immune response mechanism to combat the invading pathogens in their surroundings. Therefore, it is very important to document how sporadic pests that occasionally appear in swarms overcome surrounding natural fungal pathogens by fully exploiting their immune defense mechanisms. In the past, the transcriptome of immune response mechanisms of different pest species that appeared in the form of swarms have been well explored in insects such as ants [10,11], fall armyworms [12], hemipteran stinkbugs [13,14], potato leafhoppers [15], and termites [16,17]. The current study was aimed to fully explore the immune-related gene expression patterns evolved in the testes, ovaries, and mid-guts of the adults (males and females) of two-spotted field crickets during swarming to document for the first time a profound understanding of the highly specialized immune responsive combating strategy against natural pathogens that will be useful to develop high-quality reference transcriptomes of two-spotted field crickets for future research into the host–pathogen interactions.

## 2. Materials and Methods

### 2.1. Collection, Maintenance, and Tissue Extraction of Two-Spotted Field Crickets

The populations of two-spotted field crickets were directly collected from their outbreak areas located in the western part of the Kingdom of Saudi Arabia. The males and females of the adults of collected populations of two-spotted field crickets were separately kept at a photoperiod of 16 h light, 8 h dark under controlled temperature conditions (30 ± 0.50 °C). Male and female adults of two-spotted field crickets were separately dissected in saline in order to separate different tissues including male mid-gut, female mid-gut, male testes, and female ovaries. These target tissues were stored in 2 mL Eppendorf tubes pre-chilled with liquid nitrogen at −80 °C. Three biological replicates for each target tissue were prepared by separately extracting them from different two-spotted field crickets.

### 2.2. Construction of cDNA Libraries of Two-Spotted Field Crickets

The frozen tissues were separately ground using liquid nitrogen in a mortar and pestle. The cDNA libraries were constructed by following the previous methodology [16]. In brief, total RNA from each sample was separately extracted using TRIzol reagent (Invitrogen, Waltham, MA, USA). The quality of the extracted total RNA was evaluated by electrophoresis (1% agarose gel).

The mRNA molecules were purified using an Oligotex mRNA Mini Kit (Qiagen, Hilden, Germany), which were used as templates to synthesize the first-strand cDNA using random hexamer primers, and ultimately to synthesize second-strand cDNA libraries. In this study, twelve cDNA libraries including three males mid-guts, three females mid-guts, three males testes, and three females ovaries were prepared to construct four cDNA libraries, each with three independent biological replicates that were separately prepared by sequencing through an Illumina HiSeq^TM^ 4000 platform at MicroAnaly Gene Technologies Co., Ltd. (Wuhan, Hubei, China). These data have been made available at the NCBI Sequence Read Archive (SRA BioProject Acc. No. PRJNA647692).

### 2.3. Sequence Assembly of the cDNA Libraries of Two-Spotted Field Crickets

After filtering, the resulting clean reads were mapped to the *G. bimaculatus* genome on NCBI (Accession: PRJNA647692 submitted by the investigators) using the Hierarchical Indexing for Spliced Alignment of Transcripts, HISAT program. All reads were assembled by the Trinity assembly program (v2.8.6) in its genome-guided mode, and biological sequences were clustered to remove sequence redundancy in order to obtain the UniGene sequence set for subsequent analysis using the CD-HIT program [18].

### 2.4. Analysis of Differential Expression of Genes

The DEGs between the paired comparisons (male mid-gut versus female mid-gut; male testes versus female ovaries) were analyzed with the Cuffdiff method. DEGs were considered between four libraries when the screening threshold of the p-value for False Discovery Rate (FDR) was less than 0.05, and an absolute value of log2Ratio FC (Fold Change) for a gene was greater than 1, using R language package DESeq2 (the screening threshold is FDR (false discovery rate) < 0.05, log_2_FC (fold change) for a gene > 1 or log_2_FC < −1) [19].

### 2.5. Functional Annotation of the cDNA Libraries of Two-Spotted Field Crickets

The new transcripts were annotated via BLAST searches against the NCBI non-redundant protein database. All identified genes were quantified in terms of the expected number of fragments per kb of transcript sequence per million base pairs sequenced (FPKM) with the software program Cufflinks. The UniGenes were explored by performing annotations through the Kyoto Encyclopedia of Genes and Genomes (KEGG) [20], through a web-based interface. Immunity-related genes were accordingly classified into their main categories.

## 3. Results

### 3.1. Samples Correlation Analysis

**Figure 1:**
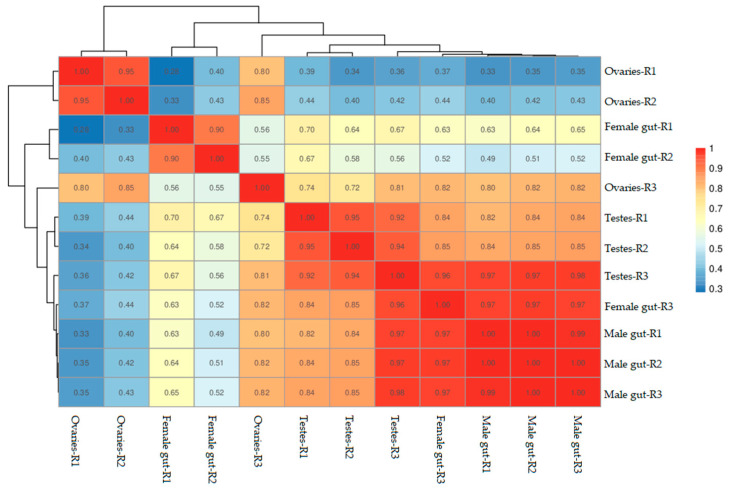
Correlations between cDNA libraries constructed from different parts including the ovaries, testes, female mid-gut, and male mid-gut of two-spotted field crickets. The correlation coefficient close to 1 represents the higher similarity of the expression patterns between samples.

The correlation of gene expression levels between samples performed in this study revealed a higher similarity of the expression patterns between samples. Among all the experimental units, biological samples collected from male mid-gut tissues were found to have the highest similarity (Figure 1). On the other hand, ovary samples have shown variability. The samples of testes have also shown similar expression patterns but remained between mid-gut and ovary samples.

### 3.2. Sequence Assembly Characteristics of Two-Spotted Field Crickets

In this study, the Illumina HiSeq platform was used to perform next-generation sequencing of two-spotted field crickets from different organs including the ovaries, testes, and male and female mid-gut samples. The RNA-Seq led to an average of 50.04 million, 74.88 million, 63.90 million, and 64.63 million clean reads for the constructed full-length cDNA libraries of male mid-gut and female mid-gut, testes, and ovaries of two-spotted field crickets, respectively (Table 1). The Illumina platform generated an average of 7.9 G, 11.77 G, 10.07 G, and 10.07 G bases of outputs of the male mid-gut, female mid-gut, testes, and ovaries libraries, respectively (Table 1). The reads were deposited on the NCBI SRA under accession SRX8826426, SRX8826427, and SRX8826430 (mid-gut of Male two-spotted field crickets); SRX8826431, SRX8826432, and SRX8826433 (mid-gut of female two-spotted field crickets); SRX8826434, SRX8826435, and SRX8826436 (testes of male two-spotted field crickets); SRX8826437, SRX8826428, and SRX8826429 (ovaries of female two-spotted field crickets).

### 3.3. Classification and Functional Annotation of Two-Spotted Field Cricket Transcriptomes

Each organ transcript assembly was completed by assembling all the three replicates of the reads and were archived into 158,658 UniGenes from the female mid-gut, 121,025 UniGenes from the male mid-gut, 157,036 UniGenes from the ovaries, and 72,216 UniGenes from the testes of two-spotted field crickets. Overall, the generated transcript sequence resources of all the studied organs were assembled into 233,172 UniGenes, which yielded approximately 163.58 million reads. Furthermore, the assembly comprised of 316.91 million reads of 325,568 transcripts. The length of the sequence reads in each case ranged between 201 to 32,390 base pairs.

**Figure 2:**
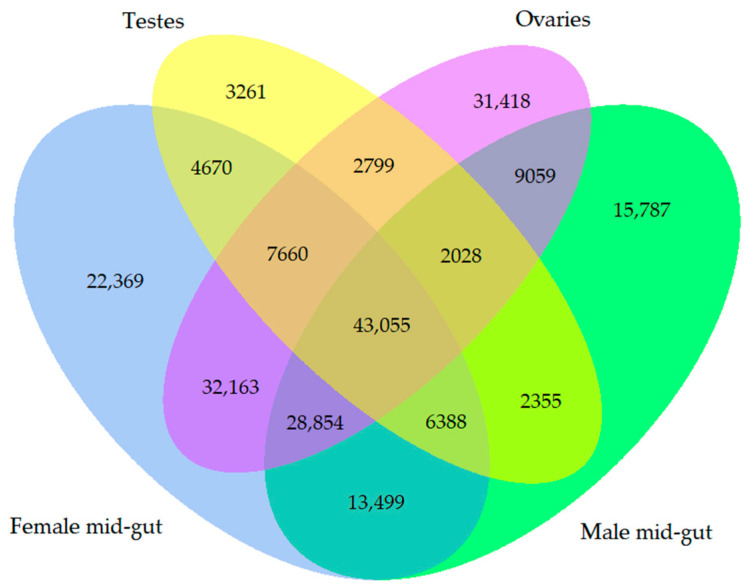
Venn diagram showing the number of overlapping genes among all the libraries of two-spotted field crickets.

The relationship of the genes assembled in this study was visualized by drawing a Venn diagram (Figure 2). Overall, 43,055 genes were common genes that were shared by all the biological samples. On the other hand, there were 15,787 unique genes from the male mid-gut, 22,369 unique genes from the female mid-gut, 31,418 unique genes from the ovaries, and 3261 unique genes from the testes that did not show any relationship.

The assembly of cDNA libraries showed variations in identified and unidentified reads among different organs. Among all the libraries, the highest reads (average of three male mid-gut libraries 22.52%) from the transcriptome of the male mid-gut were successfully identified, while the least reads (average of three Ovaries libraries 3.51%) were identified from the cDNA libraries constructed from the ovaries of two-spotted field crickets (Table 2). On the other hand, 10.28% (average of three female mid-gut libraries), and 12.40% (average of three testes libraries) assembled reads from the cDNA libraries of the female mid-gut and testes were identified, respectively. Interestingly, a major proportion of the identified reads matched with the Arthropoda, as can be seen in Table 2.

**Figure 3:**
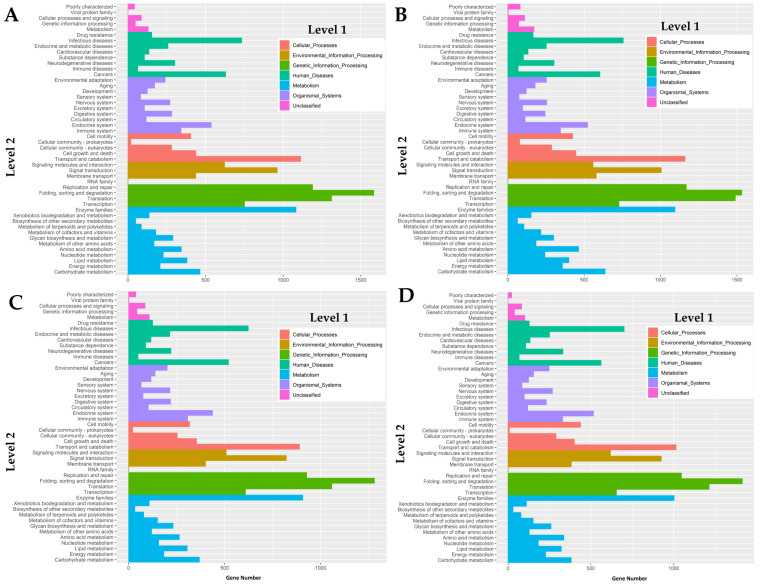
GO mapping of the cDNA libraries of (**A**) female mid-gut, (**B**) male mid-gut, (**C**) testes, and (**D**) ovaries of two-spotted field crickets by the Kyoto Encyclopedia of Genes and Genomes, KEGG classification.

The transcriptomes were successfully mapped onto the Kyoto Encyclopedia of Genes and Genomes (KEGG) pathways in order to reveal biochemical pathways operating in two-spotted field crickets. Overall, the KEGG analysis revealed the seven different main functional processes (level 1), which were further composed of 48 GO terms as shown in Figure 3. Based on the analysis, a great number of genes mapped in this analysis were involved in the regulation of host defense mechanisms through signal transduction and immune system GO terms among all the cDNA libraries.

### 3.4. Patterns of Differential Gene Expression Levels

Cluster analysis of gene expression patterns can intuitively reflect the level of gene expression and expression patterns in multiple samples. Overall, 2242 UniGenes were up-regulated, while 1805 were down-regulated between the male and female mid-gut samples. On the other hand, a comparatively a higher number of UniGenes were up-related (8921), and concurrently a higher number of genes were also down-regulated (8716) between teste and ovary samples of the two-spotted field crickets. 

### 3.5. Immune-Related Transcriptome of Two-Spotted Field Crickets

The transcriptome of two-spotted field crickets assembled as a result of the current study revealed the identification of 492 different types of genes regulating the immune response mechanism under natural conditions (Supplementary Tables S1–S5). The immunity-related transcript analysis successfully identified a wide range of Pattern Recognition Receptors (62 genes), most importantly *PRPs*, *CTLs*, *GALE*, *GNBP*, *Immulectin*, *Beta-1*, *3-glucan-binding protein*, and *DSCAM*, which actually initiate the host immune defense mechanism (Table 3).

We identified 57 genes amplifying the pathogen invasive signals through signal modulation. The representatives of these genes including *ATs*, *Ast*, *Ang*, *Chymotrypsin*, *Kazal domain-containing peptide*, *Kunitz-type protease inhibitor*, *Porin*, *proPO*, *Prostaglandin*, *SPs*, *Serpin*, and *Tetraspanin*, modulate a wide range of signaling pathways to combat pathogen invasion (Table 3 and Table S2). The activated signaling pathways including the JAK-STAT signaling Pathway, JNK pathway, Toll-like receptor (TLR) signaling pathways, Wnt signaling pathway, Notch signaling pathway, Hedgehog signaling pathway, Hippo signaling pathway, Immune Deficiency (Imd) pathway, and MAPK (Mitogen-activated protein kinases) signaling pathways are regulated through the identification of 214 genes (Table S3), involved in signal transduction (Table 3). The effectors expressed as a result of the immune mechanism among two-spotted field crickets revealed the identification of 36 genes (Table S4), encoding antimicrobial peptides and proteins. However, *Attacin*, *Bacteriocin*, *Carboxypeptidase*, *Cathepsin*, *Caspase*, *Lysozymes*, *Pyocin*, and *Thaumatin-like protein* were the most prominent genes identified in this study from two-spotted field crickets (Table 3). On the other hand, our transcriptome exploration study also revealed the identification of 123 genes categorized as other immunity-related genes because their role in immune mechanism is not yet well categorized (Table S5).

## 4. Discussion

Our study to explore the molecular mechanisms of the immune system of two-spotted field crickets by next-generation sequencing of male mid-gut, female mid-gut, ovaries, and testes revealed the identification of novel genes contributing in the regulation of host defence. The characterized annotated transcriptome as a result of this study enabled us to suggest that *G. bimaculatus* displayed a strong antimicrobial response in the form of effectors to defend naturally occurring pathogens. The current findings greatly help us to understand the molecular mechanisms of host–pathogen relationships.

The transcriptome sequencing of two-spotted field crickets performed in this study from tissues mainly aimed to explore for the first time the molecular mechanism of immunity in *G. bimaculatus.* Our methodology was successfully able to assemble the genome into 233,172 UniGenes, which yielded approximately 163.58 million reads. Furthermore, the assembly yielded 316.91 million reads of 325,568 transcripts. However, results revealed the variability, especially ovaries R3, and female mid-gut R3 biological replicates. The variations among few biological replicates evidenced in the current study and strengthened by previous investigation are quite obvious due to environmental and genetic differences because each sample was prepared by separately extracting from different two-spotted field crickets [21]. Interestingly, the annotated sequences identified a huge number of genes (492) regulating the host defence mechanisms. The Pattern Recognition Receptors (PRRs) initiate the host immune defence system by recognising the receptors for pathogen-associated molecular patterns (PAMPs) [22]. Upon recognition of PAMPs, these PRRs can either mediate pathogen killing directly through phagocytosis and encapsulation or indirectly through intra-cellular signal transduction pathways. These pathways ultimately lead to the transcription of effector genes [23,24,25,26]. Our transcriptomic analysis revealed the identification of several different classes of PRRs, and most importantly *GNBP1*, *beta-1,3-glucan-binding protein*, multiple isoforms of *apolipophorin*, *Ataxin*, *CTL*, *DSCAM*, *GALE*, and *Immulectin*. These recognition receptors are bounded with the components of the microbial cell wall, and it ultimately started a tug-of-war between the host and the invading pathogen [27,28].

Our two-spotted field cricket transcriptome analysis revealed the identification of five different isoforms of the *apolipophorin family (apoLp).* The members of the *apoLp* family are known to be involved in multiple functions, especially in activating the immune response through binding with β-1,3-glucans of fungi, LPSs of Gram-negative bacteria, and lipoteichoic acid of Gram-positive bacteria [29,30]. The previous findings of *Galleria mellonella* already showed that *apoLp* not only binds to fungal conidia and beta-1,3-glucan, but also stimulates cellular encapsulation [31]. The *apoLp* of *G. mellonella* affects the fungal cell wall components and exhibits antibacterial activity against selected gram-positive and gram-negative bacteria *in vitro* [32,33]. Our results showed five *apoLp*, which might play an important role in microbial infection. The transcriptome analysis also revealed the identification of *C-type lectins*, *Immunolectin*, *Lectin-related*, and *Galectin*, which are known to be involved in the innate immune response. They recognize the chains of polysaccharide present on the surface of pathogens [34,35]. Another class of PRRs, such as *GNBP1*, which triggered the protease cascades by recognizing gram-positive bacteria, was identified in this study and ultimately causes the cleavage of Spaetzle [36]. Such a wide range of transcripts of PRRs revealed for the first time as a result of this study from two-spotted field crickets enabled us to suggest that the host has well-developed weaponry mechanisms to recognize the invading natural pathogens prevailing in their surroundings.

Once the invading pathogen has been recognized, PRRs triggers the initiation of signal modulation genes that amplify the signals of pathogen invasion. These signals ultimately activate various lines of defence against the invasion of pathogens. In this transcriptome analysis, we identified 57 genes encoding proteins potentially involved in signal modulation. From our database, a number of signaling modulation genes were observed such as CLIP domain (CLIPs), *serine*, and *serpins*. Signal modulation genes, especially *serine proteases* (*SPs*), regulate several invertebrate defense responses, including hemolymph coagulation, antimicrobial peptide synthesis after toll signal-transduction pathway and activation of phenoloxidases (POs) [37]. Serine-type protease inhibitors (*Serpins*) and *Kazal* play an important role in inhibiting the protease cascades that activate toll and melanization reaction in *Drosophila* [23,38]. Serpin-like proteins have already been reported in many insects such as *H. cunea*, *A. melifera*, *A. gambiae*, *B. mori*, and *D. melanogaster* [39,40,41]. Interestingly, our transcriptome analysis successfully annotates genes modulating the immune mechanism.

Various pathways regulate the immune response of invertebrates against invading pathogens by transmitting signals from recognition receptors to the synthesis of AMPs and other effectors. The current exploration annotated 214 genes involved in various types of immune signaling pathways including JAK-STAT, JNK, Toll-like receptor (TLR), Wnt, Notch, Hedgehog, Hippo, Immune Deficiency (Imd), and MAPK (Mitogen-activated protein kinases) signaling pathways.

Members of the Ras superfamily identified in this transcriptome exploration are reported to be involved in the complex signaling pathways of *Drosophila* [42]. The Ras superfamily is the protein of small guanosine triphosphatases (GTPases) comprised of five major families, including *Arf/Sar*, *Rab*, *Ran*, *Ras*, and *Rho* [43,44]. The exact role of these genes in *G. bimaculatus* is unknown. However, *Ras* genes are known to be involved in the cellular immune response of beet armyworm, *S. exigua* [45]. Furthermore, Rojas., et al. [44] reviewed the role of *Ras* superfamily member genes and explained that they act as signaling nodes that regulate apoptosis, cell proliferation, and differentiation. Another important gene was the *Four-and-a-half LIM domain protein 1 isoform B.* These cysteine-rich LIM domain-containing genes are previously found to mediate signal transduction cascades. Their deficiency in mice resulted in delayed wound healing [46]. The presence of *Serine/threonine protein kinase (STK)* from the transcriptome of two-spotted field crickets is an important finding because these genes are well known to function as an important defence gene by mediating signal transduction pathways in plants [47]. Their role in the insect immune signaling pathway has been explored by Belvin and Anderson [48]. They suggested that *STK* is an important component of the Toll-Dorsal pathway responsible for the degradation of cactus proteins in *Drosophila*. The various isoforms of genes encoding *Zinc finger proteins* characterized in the current study promote the *Toll-like receptors* as depicted in the current study to trigger innate immune responses by pressing IκBα gene transcription as previously reported from human beings [49]. On the other hand, *Kruppel-like protein* characterized here is a transcriptional repressor associated with signal transduction and activator of transcription (STATs) in the immune response [50]. 

The transcriptome analysis showed the presence of *14-3-3* genes from the two-spotted field crickets. These signal transduction regulatory genes interact in a phospho-serine dependent manner. The members of this protein are involved in cellular and physiological processes. The recent findings suggested that these genes are important phagocytosis mediators that play a pivotal role in defending *S. aureus* attack on *Drosophila* and zebrafish [51]. Furthermore, they illustrated that the depletion of *14-3-3* expression reduced the ability to fight against microbial infection. However, this depletion does not compromise the production of antimicrobial peptides [51]. In addition to gene encoding *14-3-3* proteins, an important multifunctional group of genes encoding *COP9 signalosome complex subunits* was identified from two-spotted field crickets. These *COP9 signalosome complex subunits* are known to function as an important defence protein and dispensable for Toll/IL-1 activation in the fat body by mediating signal transduction pathways in *Drosophila* [52]. They suggested that *CSN5* is an important component of signaling pathways involved in Cactus and Dorsal regulation to mediate immune response in *Drosophila*.

The genes encoding *EF-hand domain-containing proteins* were also identified in this study. The members of this domain, including *calmodulins*, are known to regulate Ca^2+^ channel in vertebrates and invertebrates [53]. These genes are known to play an important role in immune responses by maintaining calcium levels. In addition to the above mentioned signal transduction genes, *Tubulin beta chain*, *Transgelin*, *Hedgehog protein*, *Hippo*, *Hippocampus abundant transcript 1 protein*, *IMD-like protein*, *JNK-interacting protein 1*, *Leucine-rich repeat-containing protein 68, Notch*, *Pelle*, *Relish*, *Renin*, *Spaetzle*, and *Wnt* identified in this study might help to understand their role in signal transduction cascades.

Our study revealed the identification of effectors among which *Attacin*, *Bacteriocin*, *thaumatin-like protein*, *C-type Lysozyme*, *I-type Lysozyme*, *Cathepsin D*, *Carboxypeptidase*, *Caspase*, and *Pyocin* were prominent. These molecules play an important direct role in combating pathogenic microorganisms. Generally, *thaumatin-like proteins* possessed 16 conserved cysteine residues that form eight disulfide bonds [54]. The antifungal activities of more than 20 isoforms of *thaumatin-like proteins* have been reported [55,56]. These proteins were first identified from the West African shrub *Thaumatococcus daniellii* and is known to synthesize in plants against stress and microbial infection [57]. Later, these proteins were further identified from animals [58], and fungi [59]. More recently, these proteins have also been identified from insects including *Acyrthosiphon pisum*, *Coptotermes formosanus* Shiraki, and *Tribolium castaneum* [16,60]. 

The transcriptome analysis of two-spotted field crickets showed multiple isoforms of several genes encoding *c-type lysozymes*, *i-type-lysozymes*, and *lysozymes*. Generally, insect lysozymes are known to play a pivotal role in insect immunity [61,62,63]. In addition, *lysozymes* isolated from the eggs and salivary glands of *Reticulitermes speratus* showed a strong novel egg recognition activity [64]. Furthermore, they observed that the newly laid eggs are frequently coated with the saliva of the worker caste containing lysozyme through egg grooming. Along with multiple isoforms of *lysozymes*, numerous genes encoding various *cathepsins* were identified. These genes have shown to regulate multiple proteins facilitating bacterial killing [65]. These proteins are known to play an important role against infections because they are believed to be highly expressed in immune-related organs [66].

Glycine-rich proteins such as *Attacin* belong to an important antimicrobial peptide group, which was for the first time discovered from *Hyalophora cecropia* [67]. The latest review article revealed the identification of different isoforms of *attacin* from different insects including silkworms, tse-tse fly, housefly, tobacco budworm, cabbage looper, and wild silkworm [68]. Our transcriptome study for the first time identified *attacin* from two-spotted field crickets. These findings enabled us to suggest the highly specialized broad spectrum host–antimicrobial response against invading pathogens.

## 5. Conclusions

In conclusion, the current study successfully constructed cDNA libraries from different tissues of the male mid-gut, female mid-gut, ovaries, and testes of two-spotted field crickets. The Illumina HiSeq platform generated an average of 7.9 G, 11.77 G, 10.07 G, and 10.07 G bases of outputs, which assembled into 121,025, 158,658, 72,216, and 157,036 UniGenes from the male mid-gut, female mid-gut, testes, and ovaries, respectively. The transcriptome analysis of the generated libraries revealed for the first time the identification of 492 different types of genes under the categories including Pattern Recognition Receptors (62 genes), Signal modulators (57 genes), Signal transduction (214), effectors (36 genes), and others (123 genes) to regulate the immune mechanism under natural conditions among two-spotted field crickets. In summary, *G. bimaculatus* transcriptome analysis provides preliminary evidence for their survival through their sophisticated and a specialized broad spectrum host immune defence mechanism against natural pathogens. It will open new avenues of research to develop molecular insecticides, and drug development by targeting genes regulating host immune defence mechanisms.

## Figures and Tables

**Table 1:** Summary of the Illumina Platform RNA-Seq Metrics for two-spotted field crickets.

Sample	NCBI SRA Accession	Biosample Accession	Clean Reads Number	No of Bases	Q20 Content (%)	Q30 Content (%)	GC Content (%)
**Mid-Gut of Male Two-Spotted Field Crickets**							
R1	SRX8826426	SAMN15594732 (SRS7091132)	42,341,558	6.7 G	93	83	38.1
R2	SRX8826427	SAMN15594733 (SRS7091133)	52,441,310	8.3 G	93	84	39.0
R3	SRX8826430	SAMN15594734 (SRS7091136)	55,325,614	8.7 G	93	84	39.2
**Mid-Gut of Female Two-Spotted Field Crickets**							
R1	SRX8826431	SAMN15594735 (SRS7091137)	88,936,348	14.0 G	94	84	49.9
R2	SRX8826432	SAMN15594736 (SRS7091138)	80,383,866	12.6 G	94	84	47.2
R3	SRX8826433	SAMN15594737 (SRS7091139)	55,325,614	8.7 G	94	85	44.3
**Testes of Male Two-Spotted Field Crickets**							
R1	SRX8826434	SAMN15594738 (SRS7091140)	67,447,566	10.6 G	94	84	49.3
R2	SRX8826435	SAMN15594739 (SRS7091141)	59,198,274	9.4 G	93	84	49.0
R3	SRX8826436	SAMN15594740 (SRS7091142)	65,058,620	10.2 G	94	85	45.2
**Ovaries of Female Two-Spotted Field Crickets**							
R1	SRX8826437	SAMN15594741(SRS7091143)	70,968,356	11.1 G	95	86	43.6
R2	SRX8826428	SAMN15594742 (SRS7091134)	58,530,318	9.1 G	95	87	41.9
R3	SRX8826429	SAMN15594743 (SRS7091135)	64,386,128	10.0 G	95	87	42.5

**Table 2:** Assembly metrics for two-spotted field crickets transcriptome sequencing.

Assembled Reads Information	Male Mid-Gut			Female Mid-Gut			Ovaries			Testes		
	R1 (%)	R2 (%)	R3 (%)	R1 (%)	R2 (%)	R3 (%)	R1 (%)	R2 (%)	R3 (%)	R1 (%)	R2 (%)	R3 (%)
Unidentified Reads	68.68	79.08	84.68	95.38	91.87	81.92	96.3	97.28	95.9	87.76	89.21	85.83
Identified Reads	31.32	20.92	15.32	4.62	8.13	18.08	3.7	2.72	4.10	12.24	10.79	14.17
Matched with Arthropoda	23.71	15.32	11.69	2.76	5.61	11.47	1.56	1.13	2.05	5.96	5.24	8.84

**Table 3:** Immune-related transcriptome of two-spotted field crickets.

Functional Categories	Characterized Annotations
Pattern Recognition Receptors	Apolipophorin, Ataxin, Beta-1,3-glucan-binding protein, C-type lectin (CTL), Down syndrome cell adhesion molecule-like protein (DSCAM), Galectin (GALE), Gram negative binding protein (GNBP), Hemolymph lipopolysaccharide-binding protein, Hemolymph juvenile hormone binding protein, Immulectin, Lectin, Peptidoglycan-recognition protein (PRP), Regenectin, Septin, Spondin, Techylectin
Signal Modulators	Allatotropin (ATs), Allatostatin (Ast), Angiopoietin (Ang), Chymotrypsin, Kazal domain-containing peptide, Kunitz-type protease inhibitor, Porin, proPO, Prostaglandin, Serine protease (SPs), Serpin, Tetraspanin
Signal Transductors	Adiponectin receptor protein, Allatostatin A prohormone, Ankyrin repeat and fibronectin type-III domain-containing protein 1, Angiomotin, Beta-arrestin2, Bursicon-beta, C2 domain-containing protein, CSN5 cop9 signalosome subunit 5, C-Jun-amino-terminal kinase-interacting protein 3, Calmodulin, Cactin, Calpain, Chimaerin, Contactin, COP9 signalosome complex, EF-hand domain-containing protein, Folliculin, Four-and-a-half LIM domain protein 1 isoform B, Frizzled, Hippo, Hippocampus abundant transcript 1 protein, IMD-like protein, JNK-interacting protein, Kruppel-like protein, Leucine-rich repeat-containing protein, Malectin-B, NACHT and WD repeat domain-containing protein 1, NACHT and Ankyrin domain protein, Nesprin, Notch, Octopamine, Pelle, Rab, Ras, Rho, Serine/threonine-protein kinase, Spaetzle, Striatin, TATA-box binding protein, Toll-like receptors, Transgelin, Transducin beta-like protein, Target of rapamycin complex 2 subunit MAPKAP1, Tubulin beta chain, WD repeat-containing protein, Wnt, WW domin protein, Zinc finger protein, 14-3-3 family protein
Effectors	Attacin, Bacteriocin, Carboxypeptidase, Cathepsin, Caspase, Lysozyme, Pyocin, C-type lysozyme, I-type lysozyme, Pyocin, Thaumatin-like protein 1

## Supplementary Materials

The following are available online at https://www.mdpi.com/2309-608X/6/4/232/s1, Table S1: Immune-related Pattern Recognition Receptors from the transcriptome of two-spotted field crickets, Table S2: Immune-related Signal Modulators from the transcriptome of two-spotted field crickets, Table S3: Immune-related Signal Transductors from the transcriptome of two-spotted field crickets, Table S4: Immune-related Effectors from the transcriptome of two-spotted field crickets, Table S5: Other Immune-related sequences from the transcriptome of two-spotted field crickets.
