# Supplementary material for: Insights into the Gryllus bimaculatus Immune-Related Transcriptomic Profiling to Combat Naturally Invading Pathogens
Source: J Fungi (Basel). 2020 Oct 18;6(4):232. doi: 10.3390/jof6040232 (PMC7711483; doi:10.3390/jof6040232)
Supplement: Supplementary file 1 [file jof-06-00232-s001.pdf]

Table S1. Immune-related Pattern Recognition Receptors from the transcriptome of two-spotted field crickets

No	Gene Sequence ID	Annotation description
1	TRINITY_DN87689_c0_g8_i1	*Apolipophorin*
2	TRINITY_DN90320_c4_g2_i2	*Apolipophorin-III*
3	TRINITY_DN83083_c0_g3_i1	*Apolipoprotein D*
4	TRINITY_DN87472_c0_g4_i2	*Apolipophorin III 1*
5	TRINITY_DN79329_c2_g3_i2	*ApolipophorinIII 3 isoform B*
6	TRINITY_DN87177_c0_g2_i15	*Ataxin-7-like protein 1*
7	TRINITY_DN69311_c0_g2_i1	*Ataxin-2-like protein*
8	TRINITY_DN79241_c0_g1_i1	*Beta-1,3-glucan-binding protein*
9	TRINITY_DN66389_c1_g1_i1	*C-type lectin 2*
10	TRINITY_DN80953_c0_g1_i1	*C-type lectin 13*
11	TRINITY_DN62874_c0_g2_i1	*Down syndrome cell adhesion molecule-like protein DSCAM2*
12	TRINITY_DN87557_c4_g2_i2	*Down syndrome cell adhesion molecule-like protein DSCAM2*
13	TRINITY_DN88698_c7_g1_i1	*Endoplasmic reticulum lectin 1*
14	TRINITY_DN81707_c0_g1_i3	*Galectin*
15	TRINITY_DN89270_c1_g1_i3	*Galectin*
16	TRINITY_DN87548_c0_g1_i1	*GNBP1*
17	TRINITY_DN88917_c0_g2_i5	*GNBP1*
18	TRINITY_DN36711_c0_g1_i1	*Hemolymph lipopolysaccharide-binding protein*
19	TRINITY_DN89574_c1_g2_i1	*Hemolymph lipopolysaccharide-binding protein*
20	TRINITY_DN84123_c0_g3_i2	*Hemolymph lipopolysaccharide-binding protein*
21	TRINITY_DN82224_c1_g1_i1	*Hemolymph lipopolysaccharide-binding protein*
22	TRINITY_DN74207_c0_g1_i1	*Hemolymph lipopolysaccharide-binding protein*
23	TRINITY_DN89591_c6_g3_i1	*Hemolymph juvenile hormone binding protein*
24	TRINITY_DN135327_c0_g1_i1	*Hemolymph lipopolysaccharide-binding protein*
25	TRINITY_DN84754_c0_g1_i1	*Hemolymph juvenile hormone binding protein*
26	TRINITY_DN86828_c2_g1_i2	*Hemolymph lipopolysaccharide-binding protein*
27	TRINITY_DN78311_c0_g1_i2	*Hemolymph lipopolysaccharide-binding protein*
28	TRINITY_DN81298_c1_g4_i1	*Hemolymph lipopolysaccharide-binding protein*
29	TRINITY_DN77376_c0_g1_i2	*Hemolymph lipopolysaccharide-binding protein*
30	TRINITY_DN86828_c1_g3_i1	*Hemolymph lipopolysaccharide-binding protein*
31	TRINITY_DN81584_c0_g1_i2	*Hemolymph lipopolysaccharide-binding protein*
32	TRINITY_DN129665_c0_g1_i1	*Hemolymph lipopolysaccharide-binding protein*
33	TRINITY_DN81273_c0_g10_i1	*Hemolymph lipopolysaccharide-binding protein*

34	TRINITY_DN86913_c0_g3_i2	*Hemolymph lipopolysaccharide-binding protein*
35	TRINITY_DN89840_c0_g1_i1	*Hemolymph lipopolysaccharide-binding protein*
36	TRINITY_DN40040_c0_g1_i1	*Hemolymph lipopolysaccharide-binding protein*
37	TRINITY_DN88441_c2_g1_i2	*Immulectin 9*
38	TRINITY_DN87890_c0_g1_i7	*Immulectin 5*
39	TRINITY_DN81407_c1_g1_i2	*Immulectin 11*
40	TRINITY_DN144682_c0_g1_i1	*Immulectin-2a*
41	TRINITY_DN87545_c2_g11_i1	*Immulectin 5*
42	TRINITY_DN84460_c2_g3_i2	*Immunolectin-A*
43	TRINITY_DN81721_c1_g1_i1	*Lectin-related protein*
44	TRINITY_DN81298_c0_g3_i1	*Lectin-related protein*
45	TRINITY_DN81584_c0_g2_i1	*Lectin-related protein (Fragment)*
46	TRINITY_DN83725_c2_g2_i1	*Peptidoglycan-recognition protein*
47	TRINITY_DN81082_c0_g1_i1	*Peptidoglycan-recognition protein LE-like Protein*
48	TRINITY_DN83462_c0_g1_i1	*Peptidoglycan-recognition protein 1*
49	TRINITY_DN80566_c0_g1_i1	*Regenectin*
50	TRINITY_DN14747_c0_g1_i1	*Regenectin*
51	TRINITY_DN87571_c3_g8_i3	*Regenectin*
52	TRINITY_DN160647_c0_g1_i1	*Putative scavenger receptor*
53	TRINITY_DN88582_c2_g1_i1	*Scavenger receptor class B member, putative*
54	TRINITY_DN85652_c0_g1_i1	*Scavenger receptor class B member 1*
55	TRINITY_DN87011_c0_g1_i1	*Scavenger receptor class B member 1*
56	TRINITY_DN84375_c5_g1_i1	*Scavenger receptor class B member 1-like*
57	TRINITY_DN74266_c0_g1_i1	*Septin*
58	TRINITY_DN89316_c1_g8_i2	*Spondin-1*
59	TRINITY_DN66908_c0_g2_i1	*Techylectin-5B*
60	TRINITY_DN81298_c2_g1_i1	*26-kDa lectin*
61	TRINITY_DN87545_c2_g16_i1	*26-kDa lectin*
62	TRINITY_DN86828_c1_g1_i1	*26-kDa lectin*

Table S2. Immune-related Signal Modulators from the transcriptome of two-spotted field crickets

No	Gene Sequence ID	Annotation description
1	TRINITY_DN90050_c0_g1_i1	*Allatotropin receptor*
2	TRINITY_DN82291_c1_g2_i1	*Allatostatin receptor*
3	TRINITY_DN85577_c0_g2_i1	*Angiopoietin-1 receptor*
4	TRINITY_DN89599_c1_g2_i1	*Chymotrypsin*
5	TRINITY_DN82706_c2_g1_i1	*Chymotrypsin*
6	TRINITY_DN89257_c2_g11_i1	*Chymotrypsin*
7	TRINITY_DN81498_c0_g2_i1	*Chymotrypsin*
8	TRINITY_DN90501_c0_g2_i2	*Chymotrypsin 12*
9	TRINITY_DN83010_c1_g5_i1	*Chymotrypsin-like protein*
10	TRINITY_DN83662_c0_g1_i3	*Chymotrypsin BI*
11	TRINITY_DN102153_c0_g1_i1	*GHKL domain-containing protein*
12	TRINITY_DN83100_c0_g2_i1	*Kazal domain-containing peptide*
13	TRINITY_DN78176_c0_g1_i1	*Kazal domain-containing peptide*
14	TRINITY_DN146932_c0_g1_i1	*Kunitz-type protease inhibitor 4 isoform B*
15	TRINITY_DN133622_c0_g1_i1	*Porin*
16	TRINITY_DN86437_c8_g1_i2	*Prophenoloxidase*
17	TRINITY_DN86437_c7_g1_i1	*Prophenoloxidase*
18	TRINITY_DN63594_c0_g1_i2	*Phenoloxidase 2-like*
19	TRINITY_DN86437_c7_g2_i2	*Pro-phenoloxidase 1*
20	TRINITY_DN87219_c10_g1_i2	*Prostaglandin E synthase 2*
21	TRINITY_DN89738_c1_g7_i6	*Serine protease 1*
22	TRINITY_DN76928_c0_g1_i1	*Serine protease 3*
23	TRINITY_DN79060_c0_g2_i2	*Serine protease*
24	TRINITY_DN129705_c0_g1_i1	*Serine protease*
25	TRINITY_DN80081_c0_g1_i1	*Serine protease*
26	TRINITY_DN82393_c0_g1_i3	*Serine protease*
27	TRINITY_DN90709_c15_g1_i1	*Serine protease*
28	TRINITY_DN84137_c0_g2_i1	*Serine protease*
29	TRINITY_DN90709_c13_g5_i1	*Serine protease*
30	TRINITY_DN86192_c2_g15_i1	*Serine protease*
31	TRINITY_DN90709_c14_g1_i1	*Serine protease*
32	TRINITY_DN78833_c0_g1_i1	*Serine protease*
33	TRINITY_DN85446_c1_g3_i1	*Serine protease*
34	TRINITY_DN86838_c0_g11_i4	*Serine protease*
35	TRINITY_DN90326_c6_g5_i1	*Serine protease*

36	TRINITY_DN82387_c0_g1_i1	*Serine protease*
37	TRINITY_DN102626_c0_g1_i1	*Serine proteinase stubble*
38	TRINITY_DN87778_c0_g1_i2	*Serine protease gd*
39	TRINITY_DN87612_c0_g5_i2	*Serine protease P69*
40	TRINITY_DN88358_c0_g1_i1	*Serine protease 62*
41	TRINITY_DN88307_c1_g2_i3	*Serine protease 14*
42	TRINITY_DN39487_c0_g1_i1	*Serine protease 26*
43	TRINITY_DN167817_c0_g1_i1	*Serine protease, S9A family peptidase*
44	TRINITY_DN78414_c0_g1_i1	*Serine protease 38*
45	TRINITY_DN89946_c0_g3_i1	*Serpin*
46	TRINITY_DN85610_c1_g1_i3	*Serpin B4*
47	TRINITY_DN88860_c0_g1_i2	*Serpin I2*
48	TRINITY_DN88170_c1_g1_i1	*Serpin protein*
49	TRINITY_DN88170_c1_g3_i3	*Serpin 5*
50	TRINITY_DN90032_c1_g3_i3	*Serpin B8*
51	TRINITY_DN89804_c1_g2_i1	*Tetraspanin*
52	TRINITY_DN88187_c4_g2_i4	*Tetraspanin*
53	TRINITY_DN88045_c3_g1_i3	*Tetraspanin*
54	TRINITY_DN81053_c1_g1_i3	*Tetraspanin*
55	TRINITY_DN61731_c0_g1_i1	*Tetraspanin*
56	TRINITY_DN166583_c0_g1_i1	*Tetraspanin*
57	TRINITY_DN79958_c0_g2_i5	*Trypsin-like serine protease*

Table S3. Immune-related Signal Transductors from the transcriptome of two-spotted field crickets

No	Gene Sequence ID	Annotation description
1	TRINITY_DN79345_c0_g1_i3	*Adiponectin receptor protein*
2	TRINITY_DN84102_c0_g1_i2	*Allatostatin A prohormone*
3	TRINITY_DN91028_c20_g3_i3	*Ankyrin repeat and fibronectin type-III domain-containing protein 1*
4	TRINITY_DN85533_c1_g2_i1	*Angiomotin*
5	TRINITY_DN90655_c0_g2_i1	*Beta-arrestin2*
6	TRINITY_DN76807_c0_g2_i1	*Bursicon-beta*
7	TRINITY_DN66564_c0_g2_i1	*C2 domain-containing protein*
8	TRINITY_DN66503_c0_g1_i1	*C2 domain protein*
9	TRINITY_DN110565_c0_g1_i1	*C2 domain protein*
10	TRINITY_DN15032_c0_g1_i1	*CSN5 cop9 signalosome subunit 5*
11	TRINITY_DN85088_c1_g1_i8	*C-Jun-amino-terminal kinase-interacting protein 3*
12	TRINITY_DN49469_c0_g1_i1	*Calmodulin-domain protein kinase*
13	TRINITY_DN129478_c0_g1_i1	*Calmodulin putative*
14	TRINITY_DN80828_c0_g1_i14	*Cactin*
15	TRINITY_DN89760_c0_g1_i5	*Calmodulin*
16	TRINITY_DN88139_c1_g1_i4	*Calmodulin-binding transcription activator 1*
17	TRINITY_DN80518_c0_g1_i2	*Calmodulin-like protein 4*
18	TRINITY_DN110393_c0_g1_i1	*Calmodulin cam-206*
19	TRINITY_DN90586_c1_g1_i9	*Calpain 1*
20	TRINITY_DN83104_c1_g2_i2	*Calpain-A*
21	TRINITY_DN86114_c0_g2_i22	*Calpain-B*
22	TRINITY_DN84320_c3_g1_i4	*Calpain-7*
23	TRINITY_DN81402_c0_g1_i3	*Calpain-A*
24	TRINITY_DN165025_c0_g1_i1	*Calpain-7*
25	TRINITY_DN86876_c0_g2_i2	*Calpain-7-like protein*
26	TRINITY_DN86519_c2_g2_i2	*Calpain-1 catalytic subunit*
27	TRINITY_DN164900_c0_g1_i1	*Calpain family cysteine protease domain-containing protein*
28	TRINITY_DN87120_c3_g4_i13	*Catenin delta-2*
29	TRINITY_DN178203_c0_g1_i1	*Chimaerin*
30	TRINITY_DN81696_c0_g1_i1	*Contactin*
31	TRINITY_DN70953_c0_g2_i1	*COP9 signalosome complex subunit 3*
32	TRINITY_DN88541_c0_g7_i1	*COP9 signalosome complex subunit 4*
33	TRINITY_DN88946_c1_g3_i4	*COP9 signalosome complex subunit 5*
34	TRINITY_DN84795_c0_g1_i1	*COP9 signalosome complex subunit 6*
35	TRINITY_DN84947_c1_g1_i2	*COP9 signalosome complex subunit 7a*

36	TRINITY_DN83545_c1_g1_i3	*COP9 signalosome complex subunit 1*
37	TRINITY_DN81987_c0_g2_i2	*COP9 signalosome complex subunit 2*
38	TRINITY_DN79343_c0_g1_i1	*EF-hand domain-containing protein 1-like*
39	TRINITY_DN85586_c1_g7_i1	*EF-hand domain containing protein*
40	TRINITY_DN81079_c0_g1_i1	*EF-hand calcium-binding domain-containing protein 2*
41	TRINITY_DN15974_c0_g1_i1	*EF hand domain-containing protein, putative*
42	TRINITY_DN32770_c0_g1_i1	*EF hand domain-containing protein*
43	TRINITY_DN84200_c1_g2_i2	*EF-hand calcium-binding domain-containing protein 4B*
44	TRINITY_DN93947_c0_g1_i1	*EF hand domain-containing protein*
45	TRINITY_DN32770_c0_g2_i1	*EF hand domain-containing protein*
46	TRINITY_DN161113_c0_g1_i1	*EF hand domain-containing protein*
47	TRINITY_DN23059_c0_g1_i1	*EF hand domain-containing protein*
48	TRINITY_DN82619_c0_g1_i2	*EF-hand domain-containing family member C2*
49	TRINITY_DN78934_c0_g2_i1	*Folliculin*
50	TRINITY_DN87350_c4_g6_i4	*Four-and-a-half LIM domain protein 1 isoform B*
51	TRINITY_DN88862_c4_g7_i1	*Frizzled-7*
52	TRINITY_DN177906_c0_g1_i1	*Frizzled-10-B*
53	TRINITY_DN81654_c0_g1_i2	*Frizzled-related protein*
54	TRINITY_DN85257_c0_g2_i2	*Gamma-tubulin complex component*
55	TRINITY_DN79544_c0_g1_i1	*Hedgehog protein*
56	TRINITY_DN89592_c1_g3_i2	*Hippo*
57	TRINITY_DN84269_c7_g7_i1	*Hippocampus abundant transcript 1 protein*
58	TRINITY_DN90160_c6_g2_i2	*IMD-like protein*
59	TRINITY_DN89357_c0_g4_i1	*JNK-interacting protein 1*
60	TRINITY_DN81682_c0_g1_i2	*JNK-interacting protein 1*
61	TRINITY_DN81843_c0_g3_i1	*JNK1-associated membrane protein*
62	TRINITY_DN88444_c11_g1_i1	*Kruppel-like protein 1*
63	TRINITY_DN89472_c0_g3_i3	*Leucine-rich repeat-containing protein 68*
64	TRINITY_DN88837_c3_g7_i1	*Leucine-rich repeat and fibronectin type III domain-containing protein 1*
65	TRINITY_DN86086_c2_g2_i2	*Leucine-rich repeat-containing protein*
66	TRINITY_DN88614_c0_g4_i2	*Leucine-rich repeat-containing protein 57*
67	TRINITY_DN82320_c0_g1_i1	*Leucine-rich repeat protein soc-2*
68	TRINITY_DN80787_c0_g1_i1	*Malectin-B*
69	TRINITY_DN89144_c3_g2_i1	*NACHT and WD repeat domain-containing protein 1*
70	TRINITY_DN77145_c1_g1_i1	*NACHT and Ankyrin domain protein*
71	TRINITY_DN89726_c0_g15_i3	*Nesprin-1*

72	TRINITY_DN82120_c0_g1_i2	*Notch*
73	TRINITY_DN15896_c0_g1_i1	*Notch*
74	TRINITY_DN143739_c0_g1_i1	*Notch 1*
75	TRINITY_DN89037_c3_g2_i1	*Notch protein*
76	TRINITY_DN84036_c0_g1_i1	*Notch protein*
77	TRINITY_DN12748_c0_g2_i1	*Notch domain-containing protein*
78	TRINITY_DN63859_c0_g2_i1	*Nuclear cap-binding protein subunit 2*
79	TRINITY_DN113923_c0_g1_i1	*Octopamine receptor 1*
80	TRINITY_DN71072_c0_g2_i1	*Pelle*
81	TRINITY_DN117445_c0_g1_i1	*Protein kinase AKT, related*
82	TRINITY_DN81879_c1_g5_i1	*Rab11 family-interacting protein 1*
83	TRINITY_DN83448_c0_g2_i1	*Rab-like protein 3*
84	TRINITY_DN49201_c0_g2_i1	*Rab GDP dissociation inhibitor*
85	TRINITY_DN110705_c0_g1_i1	*Rab-GTPase-TBC domain protein*
86	TRINITY_DN93717_c0_g1_i1	*Ras-specific guanine nucleotide-releasing factor 2*
87	TRINITY_DN74295_c0_g1_i1	*Rab-GTPase-TBC domain protein*
88	TRINITY_DN49201_c0_g1_i1	*Rab GDP dissociation inhibitor*
89	TRINITY_DN115735_c0_g1_i1	*Rab11b*
90	TRINITY_DN129461_c0_g1_i1	*Rab GDP dissociation inhibitor*
91	TRINITY_DN77261_c0_g1_i2	*Rab-like protein 2A*
92	TRINITY_DN77455_c0_g3_i1	*Rab18-family small gtpase*
93	TRINITY_DN58433_c0_g1_i1	*Rab GDP dissociation inhibitor*
94	TRINITY_DN78860_c0_g1_i5	*Ras-related protein Rab-36*
95	TRINITY_DN77306_c0_g2_i1	*Ras-like protein family member 11B*
96	TRINITY_DN128808_c0_g1_i1	*Ras family protein*
97	TRINITY_DN90571_c3_g4_i3	*Ras-like GTP-binding protein Rho1*
98	TRINITY_DN5265_c0_g1_i1	*Ras-like protein 3*
99	TRINITY_DN84087_c2_g2_i6	*Ras-related protein Rab-27A*
100	TRINITY_DN78011_c0_g3_i1	*Ras GTPase-activating protein-binding protein 1-like isoform X1*
101	TRINITY_DN86370_c6_g1_i1	*Ras-like protein 2 protein*
102	TRINITY_DN81939_c0_g2_i1	*Ras association domain-containing protein 10*
103	TRINITY_DN77306_c0_g1_i1	*Ras-like protein family member 11B*
104	TRINITY_DN78011_c0_g2_i1	*Ras GTPase-activating protein-binding protein 1-like isoform X1*
105	TRINITY_DN60273_c0_g1_i1	*Ras small GTpase, putative*
106	TRINITY_DN70163_c0_g1_i3	*Ras-specific guanine nucleotide-releasing factor 2*
107	TRINITY_DN87176_c0_g1_i1	*Ras-associated and pleckstrin-like proteiny domains-containing protein 1*

108	TRINITY_DN87642_c1_g1_i11	*Ras GTPase-activating protein 1*
109	TRINITY_DN76002_c0_g1_i1	*Ras suppressor protein 1*
110	TRINITY_DN81692_c0_g1_i2	*Ras-related protein Rab-8A*
111	TRINITY_DN89075_c2_g4_i9	*Ras gtpase-activating protein isoform x1*
112	TRINITY_DN79802_c0_g1_i1	*Ras GTPase-activating protein 3*
113	TRINITY_DN81323_c1_g3_i1	*Relish*
114	TRINITY_DN80870_c0_g2_i2	*Renin receptor*
115	TRINITY_DN84598_c0_g1_i1	*Rho GTPase-activating protein 18*
116	TRINITY_DN90173_c1_g1_i1	*Rho-associated protein kinase 2*
117	TRINITY_DN87785_c1_g2_i1	*Rho GTPase-activating protein 17*
118	TRINITY_DN86960_c5_g3_i3	*Rho GTPase-activating protein 20*
119	TRINITY_DN80784_c0_g1_i1	*Rho GTPase-activating protein 12*
120	TRINITY_DN3965_c0_g1_i1	*Rho GTPase-activating protein 20*
121	TRINITY_DN89028_c1_g1_i10	*Roquin*
122	TRINITY_DN86150_c4_g5_i7	*Serine-protein kinase*
123	TRINITY_DN104362_c0_g1_i1	*Serine protein kinase RIO*
124	TRINITY_DN3140_c0_g2_i1	*Serine/threonine protein kinase*
125	TRINITY_DN86137_c7_g1_i13	*Serine/threonine-protein kinase ULK3*
126	TRINITY_DN84829_c0_g1_i1	*Serine/threonine-protein kinase receptor*
127	TRINITY_DN74470_c0_g2_i1	*Serine/threonine-protein phosphatase*
128	TRINITY_DN90497_c2_g3_i6	*Serine/threonine-protein kinase/endoribonuclease IRE2*
129	TRINITY_DN90556_c0_g1_i2	*Serine/threonine-protein kinase 36*
130	TRINITY_DN129044_c0_g1_i1	*Serine/threonine-protein phosphatase*
131	TRINITY_DN90593_c2_g3_i2	*serine/threonine-protein kinase Chk2 isoform X2*
132	TRINITY_DN83761_c1_g1_i2	*Serine/threonine-protein kinase D3*
133	TRINITY_DN18171_c0_g2_i1	*Serine/threonine-protein phosphatase*
134	TRINITY_DN99335_c0_g1_i1	*Serine/threonine transporter SstT*
135	TRINITY_DN86442_c0_g2_i2	*Serine/threonine-protein phosphatase*
136	TRINITY_DN168219_c0_g1_i1	*Serine/threonine protein kinase*
137	TRINITY_DN87983_c1_g1_i1	*Serine/threonine-protein phosphatase 6 regulatory ankyrin repeat subunit B*
138	TRINITY_DN84921_c0_g1_i1	*Serine/threonine-protein kinase TBK1*
139	TRINITY_DN85971_c4_g2_i1	*Serine/threonine-protein phosphatase 2A regulatory subunit B′′ subunit alpha*
140	TRINITY_DN120489_c0_g1_i1	*Serine/threonine-protein kinase*
141	TRINITY_DN89738_c0_g3_i6	*Serine/threonine-protein kinase SBK1*
142	TRINITY_DN87101_c1_g5_i6	*Serine/threonine-protein kinase PRP4-like protein*

143	TRINITY_DN140017_c0_g1_i1	*Serine/threonine-protein kinase receptor*
144	TRINITY_DN87560_c2_g1_i3	*Serine/threonine-protein kinase Nek2*
145	TRINITY_DN85397_c0_g1_i1	*Serine/threonine-protein kinase PLK4*
146	TRINITY_DN90436_c1_g5_i3	*Serine/threonine-protein kinase wnk 1,3,4, putative*
147	TRINITY_DN84662_c4_g2_i7	*Serine/threonine-protein kinase SMG1*
148	TRINITY_DN79111_c0_g1_i1	*Signal transducing adapter molecule 1*
149	TRINITY_DN83589_c0_g2_i2	*Signal transducer and activator of transcription*
150	TRINITY_DN88650_c9_g2_i1	*Spaetzle*
151	TRINITY_DN83732_c1_g5_i1	*Striatin-3*
152	TRINITY_DN135560_c0_g1_i1	*TATA-box binding protein*
153	TRINITY_DN82116_c0_g1_i2	*Testis-specific serine/threonine-protein kinase 4*
154	TRINITY_DN77011_c0_g2_i1	*Toll*
155	TRINITY_DN81353_c0_g1_i1	*Toll*
156	TRINITY_DN88272_c3_g5_i1	*Toll-like receptor 4*
157	TRINITY_DN77931_c0_g1_i3	*Toll-like receptor*
158	TRINITY_DN84024_c2_g10_i1	*Toll-like recptor 8*
159	TRINITY_DN84024_c2_g5_i1	*Toll-like receptor 4*
160	TRINITY_DN84024_c2_g1_i3	*Toll-like receptor 4*
161	TRINITY_DN83352_c0_g1_i1	*Toll-interacting protein*
162	TRINITY_DN83805_c1_g5_i1	*Toll family protein 6*
163	TRINITY_DN149072_c0_g1_i1	*Toll-like receptor 4*
164	TRINITY_DN86923_c1_g1_i1	*Toll family protein 8*
165	TRINITY_DN77011_c0_g1_i1	*Toll-like receptor 1*
166	TRINITY_DN78593_c0_g1_i5	*Toll pathway, mitochondrial*
167	TRINITY_DN89445_c3_g1_i1	*Toll-like receptor 7*
168	TRINITY_DN86701_c2_g1_i5	*Toll family protein*
169	TRINITY_DN83044_c0_g1_i2	*Toll-like receptor 4*
170	TRINITY_DN89850_c4_g1_i1	*Transgelin*
171	TRINITY_DN84601_c0_g1_i1	*Transducin beta-like protein 2*
172	TRINITY_DN84168_c0_g11_i1	*Tubulin beta chain*
173	TRINITY_DN77120_c0_g1_i1	*Tubulin beta chain*
174	TRINITY_DN17472_c0_g2_i1	*Tubulin beta chain*
175	TRINITY_DN176160_c0_g1_i1	*Tubulin gamma chain*
176	TRINITY_DN90249_c0_g8_i1	*Tubulin beta chain*
177	TRINITY_DN89207_c2_g1_i1	*Tubulin beta chain*
178	TRINITY_DN84168_c0_g2_i1	*Tubulin beta chain*
179	TRINITY_DN79085_c0_g2_i6	*Tubulin alpha chain*

180	TRINITY_DN90249_c1_g5_i1	*Tubulin beta chain*
181	TRINITY_DN90255_c1_g17_i3	*Tubulin beta chain*
182	TRINITY_DN162710_c0_g1_i1	*Tubulin alpha chain*
183	TRINITY_DN187097_c0_g1_i1	*Tubulin beta chain*
184	TRINITY_DN90255_c2_g1_i1	*Tubulin beta-4 chain*
185	TRINITY_DN85145_c1_g8_i2	*Target of rapamycin complex 2 subunit MAPKAP1*
186	TRINITY_DN52654_c0_g1_i1	*Target of rapamycin complex subunit lst8*
187	TRINITY_DN167239_c0_g1_i1	*WD40 associated region in TFIID subunit*
188	TRINITY_DN83976_c0_g3_i2	*WD repeat-containing protein 48*
189	TRINITY_DN71338_c0_g1_i1	*Wnt*
190	TRINITY_DN89225_c0_g1_i1	*Wnt*
191	TRINITY_DN86967_c0_g1_i1	*Wnt*
192	TRINITY_DN87582_c0_g1_i2	*Wnt*
193	TRINITY_DN109037_c0_g1_i1	*WW domain protein*
194	TRINITY_DN169912_c0_g1_i1	*WW domain protein*
195	TRINITY_DN90343_c4_g1_i1	*Zinc finger protein*
196	TRINITY_DN199998_c0_g1_i1	*Zinc finger*
197	TRINITY_DN85604_c0_g1_i2	*Zinc finger MYND domain-containing protein 11*
198	TRINITY_DN183519_c0_g1_i1	*Zinc finger MYM-type protein 1*
199	TRINITY_DN86312_c2_g1_i2	*Zinc finger RNA-binding protein*
200	TRINITY_DN40205_c0_g1_i1	*zinc finger MYM-type protein 1-like isoform X1*
201	TRINITY_DN84851_c1_g1_i4	*Zinc finger CCHC domain-containing protein 2*
202	TRINITY_DN86155_c1_g2_i1	*Zinc finger protein ZIC, putative*
203	TRINITY_DN57944_c0_g1_i1	*Zinc finger protein 549*
204	TRINITY_DN86727_c0_g1_i1	*Zinc finger MYND domain-containing protein 11*
205	TRINITY_DN87198_c3_g2_i7	*Zinc finger C2HC domain-containing protein 1C-like Protein*
206	TRINITY_DN82107_c1_g3_i2	*Zinc finger SWIM domain-containing protein 7*
207	TRINITY_DN84461_c0_g2_i1	*Zinc finger MYND domain-containing protein 11*
208	TRINITY_DN20640_c0_g1_i1	*14-3-3 family protein*
209	TRINITY_DN90246_c0_g1_i2	*14-3-3 protein 1*
210	TRINITY_DN5987_c0_g1_i1	*14-3-3 protein epsilon*
211	TRINITY_DN45288_c0_g1_i1	*14-3-3 protein epsilon*
212	TRINITY_DN88819_c0_g9_i2	*14-3-3zeta_1 protein*
213	TRINITY_DN78767_c0_g2_i1	*14-3-3 protein epsilon*
214	TRINITY_DN405_c0_g1_i1	*14-3-3 family protein*

Table S4. Immune-related Effectors from the transcriptome of two-spotted field crickets

No	Gene Sequence ID	Annotation description
1	TRINITY_DN89440_c1_g2_i1	*Attacin*
2	TRINITY_DN165858_c0_g1_i1	*Bacteriocin*
3	TRINITY_DN69837_c0_g2_i1	*Carboxypeptidase*
4	TRINITY_DN86792_c2_g17_i1	*Carboxypeptidase*
5	RINITY_DN86792_c2_g10_i1	*Carboxypeptidase 3*
6	TRINITY_DN82454_c0_g1_i1	*Carboxypeptidase 4*
7	TRINITY_DN85268_c4_g6_i1	*Carboxypeptidase B-like*
8	TRINITY_DN88735_c2_g2_i4	*Carboxypeptidase E*
9	TRINITY_DN87822_c2_g3_i3	*Carboxypeptidase M*
10	TRINITY_DN201489_c0_g1_i1	*Carboxypeptidase yscS*
11	TRINITY_DN89787_c1_g9_i1	*Zinc carboxypeptidase*
12	TRINITY_DN88701_c6_g7_i1	*Zinc carboxypeptidase A 1*
13	TRINITY_DN195416_c0_g1_i1	*Serine-type D-Ala-D-Ala carboxypeptidase*
14	TRINITY_DN89725_c1_g1_i7	*Cathepsin 5*
15	TRINITY_DN86640_c0_g1_i2	*Cathepsin L-like protein*
16	TRINITY_DN84880_c3_g1_i6	*Cathepsin 4*
17	TRINITY_DN18659_c0_g1_i1	*Cathepsin B2*
18	TRINITY_DN146289_c0_g1_i1	*Cadherin-89D*
19	TRINITY_DN60654_c0_g1_i1	*Cathepsin L-like cysteine protease*
20	TRINITY_DN128294_c0_g1_i1	*Cathepsin L-like cysteine protease*
21	TRINITY_DN86630_c4_g2_i4	*Caspase-1 isoform X1*
22	TRINITY_DN83280_c0_g1_i1	*Caspase-1*
23	TRINITY_DN88257_c3_g6_i2	*Caspase-8*
24	TRINITY_DN85092_c0_g1_i2	*Caspase-8*
25	TRINITY_DN84211_c0_g1_i2	*Caspase-1*
26	TRINITY_DN83904_c0_g3_i4	*Caspase-1*
27	TRINITY_DN111239_c0_g1_i1	*Metacaspase*
28	TRINITY_DN163248_c0_g1_i1	*Lysozyme*
29	TRINITY_DN35342_c0_g1_i1	*Lysozyme*
30	TRINITY_DN156575_c0_g1_i1	*Lysozyme*
31	TRINITY_DN75097_c0_g1_i1	*C-type lysozyme*
32	TRINITY_DN88762_c0_g6_i1	*I-type lysozyme 1*
33	TRINITY_DN89187_c1_g12_i1	*I-type lysozyme*
34	TRINITY_DN88762_c0_g5_i1	*I-type lysozyme 1*
35	TRINITY_DN151148_c0_g1_i1	*Pyocin*

36	TRINITY_DN86520_c0_g1_i1	*Thaumatin-like protein 1*

Table S5. Other Immune-related sequences from the transcriptome of two-spotted field crickets

No	Gene Sequence ID	Annotation description
1	TRINITY_DN72364_c0_g1_i1	*Aminopeptidase*
2	TRINITY_DN116728_c0_g1_i1	*Aminopeptidase*
3	TRINITY_DN87804_c3_g1_i1	*Aminopeptidase*
4	TRINITY_DN132310_c0_g1_i1	*Aminopeptidase*
5	TRINITY_DN116492_c0_g1_i1	*Aminopeptidase N*
6	TRINITY_DN87994_c0_g1_i3	*Aminopeptidase*
7	TRINITY_DN14386_c0_g2_i1	*Aminopeptidase N*
8	TRINITY_DN87752_c0_g1_i2	*Aminopeptidase*
9	TRINITY_DN88671_c2_g3_i2	*Aminopeptidase*
10	TRINITY_DN43131_c0_g1_i1	*Aminopeptidase*
11	TRINITY_DN121703_c0_g1_i1	*Aminopeptidase N*
12	TRINITY_DN84334_c0_g1_i3	*Annexin*
13	TRINITY_DN89843_c0_g3_i8	*Annexin*
14	TRINITY_DN89702_c0_g1_i8	*Anoctamin*
15	TRINITY_DN161265_c0_g1_i1	*Anoctamin*
16	TRINITY_DN40466_c0_g2_i1	*Anoctamin*
17	TRINITY_DN90136_c2_g2_i2	*Aprataxin and PNK-like factor*
18	TRINITY_DN83533_c0_g2_i1	*Arrestin domain-containing protein 2*
19	TRINITY_DN71218_c0_g1_i2	*Arrestin1*
20	TRINITY_DN88258_c0_g8_i1	*Atlastin*
21	TRINITY_DN56888_c0_g1_i1	*Bacterial Ig-like domain protein*
22	TRINITY_DN86956_c3_g1_i5	*Basigin*
23	TRINITY_DN79324_c0_g1_i1	*Battenin*
24	TRINITY_DN79328_c0_g8_i1	*Beta-globin*
25	TRINITY_DN83003_c0_g1_i1	*Bleomycin hydrolase*
26	TRINITY_DN86495_c2_g1_i5	*Calsyntenin-1*
27	TRINITY_DN98401_c0_g1_i1	*Colicin I receptor*
28	TRINITY_DN111001_c0_g1_i1	*Colicin I receptor*
29	TRINITY_DN142439_c0_g1_i1	*Ficolin-1*
30	TRINITY_DN88733_c5_g2_i1	*FK506-binding protein 4*
31	TRINITY_DN83337_c0_g1_i3	*FK506-binding protein*
32	TRINITY_DN86026_c2_g2_i3	*Gelsolin, cytoplasmic*

33	TRINITY_DN90574_c1_g1_i2	*Gephyrin*
34	TRINITY_DN81569_c0_g1_i3	*GILT-like protein C02D5.2*
35	TRINITY_DN88839_c1_g6_i2	*Heat shock protein 90*
36	TRINITY_DN74518_c0_g3_i2	*Heat shock 70 kDa protein cognate 2*
37	TRINITY_DN57888_c0_g1_i1	*Heat shock protein 70*
38	TRINITY_DN86611_c1_g3_i1	*60 kDa heat shock protein, mitochondrial*
39	TRINITY_DN88523_c2_g1_i2	*Heat shock 70 kDa protein 14*
40	TRINITY_DN86383_c0_g5_i3	*Heat shock 70 kDa protein 12A*
41	TRINITY_DN183465_c0_g1_i1	*Heat shock protein HSP60*
42	TRINITY_DN93491_c0_g1_i1	*Activator of Hsp90 ATPase-like protein*
43	TRINITY_DN90054_c2_g3_i1	*Heat shock 70 kDa protein cognate 4*
44	TRINITY_DN81618_c1_g2_i4	*Heat shock cognate 70 kDa protein*
45	TRINITY_DN114931_c0_g1_i1	*HSP90*
46	TRINITY_DN121344_c0_g1_i1	*Hsp78p*
47	TRINITY_DN72897_c0_g4_i1	*Heat shock protein 70*
48	TRINITY_DN176147_c0_g1_i1	*Heat shock protein 90*
49	TRINITY_DN26383_c0_g2_i1	*Heat shock protein SSC1, mitochondrial*
50	TRINITY_DN68944_c0_g1_i1	*Heat shock protein beta-11*
51	TRINITY_DN57888_c1_g1_i1	*Heat shock protein SSA1*
52	TRINITY_DN157740_c0_g1_i1	*HSP70 family*
53	TRINITY_DN82806_c0_g1_i1	*Heat shock protein 70*
54	TRINITY_DN89622_c0_g3_i2	*Heat shock 70 kDa protein 4L*
55	TRINITY_DN183471_c0_g1_i1	*Heat shock protein 70*
56	TRINITY_DN85656_c0_g2_i3	*Heat shock 70 kDa protein cognate*
57	TRINITY_DN105459_c0_g1_i1	*HSP90-domain-containing protein*
58	TRINITY_DN171774_c0_g1_i1	*Heat shock protein 60*
59	TRINITY_DN89864_c3_g3_i4	*Heat shock protein 67B2*
60	TRINITY_DN146233_c0_g1_i1	*Heat shock protein*
61	TRINITY_DN134020_c0_g1_i1	*Heat shock protein 90*
62	TRINITY_DN43371_c0_g1_i1	*Heat shock protein*
63	TRINITY_DN87035_c1_g1_i1	*Heat shock factor 2-binding protein*
64	TRINITY_DN88273_c5_g1_i3	*Heat shock protein 20.7*
65	TRINITY_DN130555_c0_g1_i1	*Heat shock protein 70*
66	TRINITY_DN89516_c0_g2_i1	*Heat shock 70 kDa protein cognate 2*
67	TRINITY_DN72897_c0_g2_i1	*Heat shock protein 70*
68	TRINITY_DN90054_c2_g1_i1	*Heat shock 70 kDa protein cognate 4*
69	TRINITY_DN153276_c0_g1_i1	*Heat shock protein 70, putative*

70	TRINITY_DN91591_c0_g1_i1	*Heat shock protein 70*
71	TRINITY_DN200570_c0_g1_i1	*30 kDa heat shock protein*
72	TRINITY_DN72897_c0_g1_i1	*Heat shock protein HSP70*
73	TRINITY_DN90349_c6_g1_i1	*Heat shock cognate 70 protein*
74	TRINITY_DN67048_c0_g1_i1	*Heat shock protein 40*
75	TRINITY_DN40795_c0_g1_i1	*Heat shock 70 kDa protein, putative*
76	TRINITY_DN68944_c0_g2_i1	*Heat shock protein beta-11*
77	TRINITY_DN74471_c0_g1_i1	*Heat shock protein SSB1*
78	TRINITY_DN79478_c0_g4_i1	*Heat shock protein 90*
79	TRINITY_DN139926_c0_g1_i1	*Heat shock protein 70*
80	TRINITY_DN177381_c0_g1_i1	*Heat shock protein 70*
81	TRINITY_DN89516_c0_g3_i1	*Heat shock protein 70*
82	TRINITY_DN83196_c0_g1_i2	*Heat shock protein 75 kDa, mitochondrial*
83	TRINITY_DN101475_c0_g1_i1	*Heat shock 70 cb*
84	TRINITY_DN66009_c0_g2_i1	*Hemicentin 1*
85	TRINITY_DN89286_c0_g1_i4	*Hemicentin-1-like protein*
86	TRINITY_DN89286_c0_g4_i1	*Hemicentin-1 (Fragment)*
87	TRINITY_DN90764_c0_g1_i8	*Heparanase*
88	TRINITY_DN86745_c0_g1_i2	*Hexamerin 1*
89	TRINITY_DN86745_c0_g2_i1	*Hexamerin 1*
90	TRINITY_DN88109_c4_g4_i1	*Iporin*
91	TRINITY_DN58810_c0_g1_i1	*Lustrin variant 1*
92	TRINITY_DN85628_c0_g3_i1	*Lustrin A*
93	TRINITY_DN70585_c0_g2_i1	*Opticin*
94	TRINITY_DN82884_c0_g1_i6	*Melanization protease 1*
95	TRINITY_DN62055_c0_g1_i2	*Natterin-3*
96	TRINITY_DN77329_c2_g3_i1	*Neprilysin-11*
97	TRINITY_DN77329_c2_g2_i1	*Neprilysin-11*
98	TRINITY_DN90456_c0_g1_i1	*Netrin receptor UNC5C*
99	TRINITY_DN89260_c2_g4_i4	*Neurocalcin*
100	TRINITY_DN87371_c1_g2_i2	*Ninjurin-2 isoform X1*
101	TRINITY_DN195667_c0_g1_i1	*Optineurin*
102	TRINITY_DN97825_c0_g1_i1	*Patatin-like phospholipase family protein*
103	TRINITY_DN85093_c0_g1_i5	*Patatin-like phospholipase domain-containing protein 2*
104	TRINITY_DN92660_c0_g1_i1	*Phosducin*
105	TRINITY_DN68809_c0_g1_i1	*Plexin-A4*
106	TRINITY_DN88784_c0_g1_i12	*Presenilin*

107	TRINITY_DN90241_c0_g4_i1	*Pumilio domain-containing protein C14orf21*
108	TRINITY_DN87015_c3_g1_i1	*Regucalcin*
109	TRINITY_DN85569_c0_g2_i1	*Regucalcin*
110	TRINITY_DN83849_c0_g1_i1	*Regucalcin*
111	TRINITY_DN86515_c4_g2_i2	*Snare protein syntaxin 1*
112	TRINITY_DN88343_c0_g2_i4	*Semaphorin-1A*
113	TRINITY_DN86843_c0_g1_i5	*Semaphorin 2a*
114	RINITY_DN83539_c0_g1_i1	*Semaphorin 1a*
115	TRINITY_DN87102_c0_g1_i3	*Syndecan*
116	TRINITY_DN83808_c2_g3_i1	*Stathmin*
117	TRINITY_DN87541_c1_g1_i1	*Syntaxin-17*
118	TRINITY_DN89678_c2_g1_i1	*Syntaxin-12*
119	TRINITY_DN92370_c0_g1_i1	*Syntaxin*
120	TRINITY_DN83951_c1_g1_i1	*Syntaxin-16*
121	TRINITY_DN84801_c0_g4_i1	*Syntaxin-6*
122	TRINITY_DN166047_c0_g1_i1	*Toxin-antitoxin system YwqK family antitoxin*
123	TRINITY_DN86439_c1_g1_i1	*Torso-like protein*
